# Cysteine immobilisation on the polyethylene terephthalate surfaces and its effect on the haemocompatibility

**DOI:** 10.1038/s41598-019-53108-2

**Published:** 2019-11-13

**Authors:** Balaji Ramachandran, Vignesh Muthuvijayan

**Affiliations:** Department of Biotechnology, Bhupat and Jyoti Mehta School of Biosciences, Indian Institute of Technology Madras, Chennai, India

**Keywords:** Biomedical materials, Implants

## Abstract

Nitric oxide (NO) is an important signalling molecule involved in haemostasis. NO, present as endogenous S-nitrosothiols, is released by cysteine through a transnitrosation reaction. To exploit this mechanism, cysteine was immobilised onto the different carboxylated polyethylene terephthalate (PET) surfaces using 1-step EDC (1-ethyl-3-(3-dimethylaminopropyl) carbodiimide) crosslinking mechanism. Immobilised cysteine concentration and NO release were dependent on the surface carboxyl density. Stability studies showed that the immobilised cysteine concentration and NO release reduced within 6 h. Immobilisation of cysteine derivatives eliminated the possibility of formation of polycysteine and its electrostatic interaction with the carboxylated PET. The immobilised cysteine concentration did not recover after DTT treatment, eliminating the possibility of disulphide bond formation. Further, cysteine was immobilised using a 2-step EDC crosslinking mechanism. Although the cysteine concentration reduced during stability studies, it recovered upon DTT treatment, indicating that cysteine forms amide bonds with the carboxylated PET and the observed decrease in cysteine concentration is probably due to the formation of disulphide bonds. The haemocompatibility of the cysteine immobilised PET surfaces showed similar results compared to the carboxylated PET. The loss of thiol groups due to the disulphide bond restricts the transnitrosation reaction. Hence, these materials can be used primarily in short-term applications.

## Introduction

Nitric oxide (NO) is one of the important signalling molecules with a wide range of biological functions. NO plays a vital physiological role in regulating vasodilation, haemostasis, neurotransmission, and immune responses^1,2^. NO is one of the early thromboregulators exhibiting inhibitory effects on platelet adhesion and aggregation^3^. NO can also diffuse readily to the surrounding platelets and continue its function^4^. In addition to these properties, nitric oxide also exhibits an inhibitory effect against the proliferation of smooth muscle cells^5,6^ and anti-bacterial activity^7^. These can have a significant role in developing materials that avoid restenosis caused by neointimal hyperplasia^8^ and implant-associated infections^9^. In the biological systems, the half-life of NO is in the order of a few seconds^10^. Hence, the effects of NO are limited to the immediate surrounding of the release area, and thereby avoiding systemic toxicity concerns. These vast roles of NO make it a promising candidate for developing non-thrombogenic surfaces.

NO-based strategies have been explored as a potential method for modifying the materials to improve the blood compatibility^11,12^. The majority of the NO-based enhancement of haemocompatibility utilises diazeniumdiolates (also known as NONOates) and S-nitrosothiols (RSNOs)^13^. Depending on the method of material modification, the duration of NO release from diazeniumdialates can vary from 10 h to 150 h^14,15^. S-nitrosothiols are more biocompatible than NONOates and release NO when the bond between the thiol/sulfhydryl group and the NO is cleaved. Different derivatives of these RSNOs have been studied for the sustainable release of NO to inhibit platelet adhesion and aggregation^16,17^. Despite observing enhanced haemocompatibility, the two major limitations in utilising these NO generating/releasing molecules for the biomedical applications are limited availability of NO and uncontrolled NO release rates^18^. Studies have shown that the stimulated endothelial cells have a NO flux of about 0.1 nmol cm^−2^ min^−1^ ^19^. Thus, the developed materials should have similar NO fluxes, for effectively inhibiting platelet adhesion onto their surfaces.

To overcome these limitations, the next generation of NO-releasing materials targeted to utilise endogenous NO donors. The chemical mechanism for releasing NO from RSNOs is by a reaction called transnitrosation. It involves the reversible transfer of the nitroso group from the RSNOs to another free thiol group^20^.

$$RSNO+R^{\prime} SH\leftrightharpoons RSH+R^{\prime} SNO$$

This mechanism can be used as a potential solution for utilising the endogenous RSNOs, (S-nitrosoalbumin and S-nitrosoglutathione) as the NO donor from the bloodstream^21^. The free thiol-containing groups such as cysteine, glutathione, human serum albumin have shown the successful transfer of NO from RSNOs^22^. Scharfstein *et al*. demonstrated that the transfer of NO occurs from high molecular weight thiols to low molecular weight thiols under *in vivo* conditions^23^. Cysteine is one of the commonly used lower molecule weight thiols for transfer of NO from the endogenous NO donors. Earlier studies have shown that attachment of cysteine significantly decreases the platelet adhesion on the polyurethane and polyethylene terephthalate surfaces through endogenous NO release^24,25^. These studies had used aminolysis for surface functionalisation and glutaraldehyde for crosslinking. As aminolysis can lead to loss of mechanical strength and glutaraldehyde crosslinking has a risk of cytotoxicity^26,27^, other surface modification strategies need to be developed for cysteine immobilisation. To overcome these limitations, cysteine was covalently attached to the carboxylated PET using EDC crosslinking^28^. However, Muthuvijayan *et al*. observed that the immobilised cysteine concentration reduced with time. It was proposed that this reduction could be due to cysteine being immobilised by thioester bonds instead of amide bonds. Besides, Muthuvijayan *et al*. had not performed *in vitro* blood studies to evaluate the haemocompatibility of the modified material. Hence, we set out to address these shortcomings by developing a stable cysteine immobilisation technique and studying the effect of cysteine attachment on haemocompatibility.

In this work, we are immobilising cysteine on the different carboxylated PET surfaces, which were reported in our previous work^29^. EDC was used as the crosslinker to form a stable amide bond between the amine group in cysteine and the carboxyl group of the modified PET. The attachment of cysteine was confirmed by Ellman’s assay. The NO release by the transnitrosation reaction was estimated by Griess assay. The stability of the cysteine attachment and the release of NO through the transnitrosation reaction were also studied. Cysteine was immobilised using two methods of EDC crosslinking to achieve covalent attachment. The surface properties of these modified polymers were evaluated using SEM, EDAX and water contact angle measurement. Finally, the effect of cysteine immobilisation on haemocompatibility was studied using platelet adhesion studies, haemolysis and whole blood analysis.

## Results

### Estimation of cysteine immobilisation and NO release

**Figure 1:**
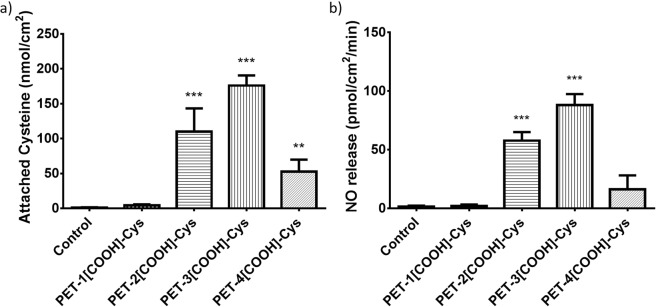
(**a**) Estimation of cysteine concentration on unmodified and cysteine immobilised PET (PET-[COOH]-Cys) using Ellman’s assay. (**b**) Estimation of nitric oxide release on unmodified and cysteine immobilised PET (PET-[COOH]-Cys) using Griess assay.

The surfaces of the PET were carboxylated using four different methods of surface modification^29^. Cysteine was immobilised onto the unmodified and carboxylated PET surfaces using 1-ethyl-3-(3-dimethylaminopropyl) carbodiimide (EDC) (1-step EDC crosslinking) to form the control, PET-1[COOH]-Cys, PET-2[COOH]-Cys, PET-3[COOH]-Cys, and PET-4[COOH]-Cys. The cysteine immobilisation was performed using 0.1 M EDC and 0.3 M cysteine at pH 6.8 and room temperature. The amount of immobilised cysteine, which was measured using Ellman’s assay, depends on the carboxyl group density on the modified PET surfaces (Fig. 1a). The mean cysteine concentration measured on the control, PET-1[COOH]-Cys, PET-2[COOH]-Cys, PET-3[COOH]-Cys, PET-4[COOH]-Cys are 1.1, 4.3, 109.9, 176.0, and 52.7 nmol/cm^2^, respectively. The PET-3[COOH], with the maximum carboxyl density, shows the maximum cysteine attachment, followed by PET-2[COOH], PET-4[COOH], and PET-1[COOH]. As Ellman’s assay quantifies only the free thiol groups, the results show that there was almost no cysteine on the unmodified PET (control) surfaces.

In the presence of nitrosothiols, cysteine takes part in a transnitrosation reaction, which involves the reversible transfer of nitric oxide (NO).

$$Donor\,[SNO]+Cys[SH]\leftrightharpoons Cys[SNO]+Donor\,[SH]$$

Griess assay estimates the nitrite (${{\rm{NO}}}_{2}^{-}$) through the azo coupling mechanism. Hence, NO release by cysteine is estimated indirectly using Griess assay. Sodium nitrite is supplied as the substrate, which releases NO under acidic conditions. NO release is estimated by the change in the nitrite concentration. On contact with PET-[COOH]-Cys films, there is a significant increase in NO release compared to control, suggesting successful transnitrosation in the presence of immobilised cysteine (Fig. 1b). The mean NO release from the control, PET-1[COOH]-Cys, PET-2[COOH]-Cys, PET-3[COOH]-Cys, PET-4[COOH]-Cys are 1.5, 2.1, 57.8, 88.1, and 16.2 pmol.cm^−2^ min^−1^, respectively. The amount of NO released correlates to the amount of free thiol group on the cysteine immobilised PET.

### Stability of thiol group of the immobilised cysteine and NO release

**Figure 2:**
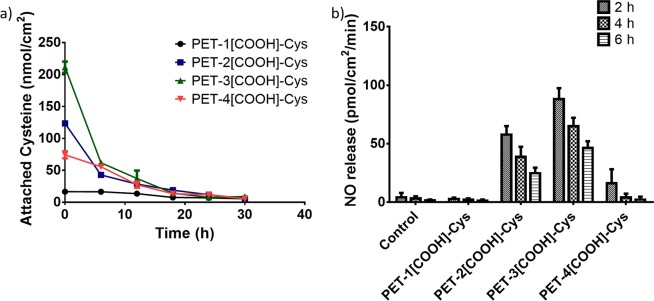
(**a**) Stability of immobilised cysteine and (**b**) NO release by transnitrosation.

The stability of the immobilised cysteine on different carboxylated PET samples was estimated in PBS (pH = 7.4) for prolonged hours. When stored in PBS at 37 °C, there was an exponential decrease in the measured cysteine concentration in all the PET-[COOH]-Cys surfaces (Fig. 2a). This is also accompanied by the reduction of NO release by transnitrosation over the period (Fig. 2b). Both the cysteine measurement by Ellman’s assay and NO estimation by Griess assay confirm the reduction in free thiol groups, which are required for the transnitrosation reaction.

**Figure 3:**
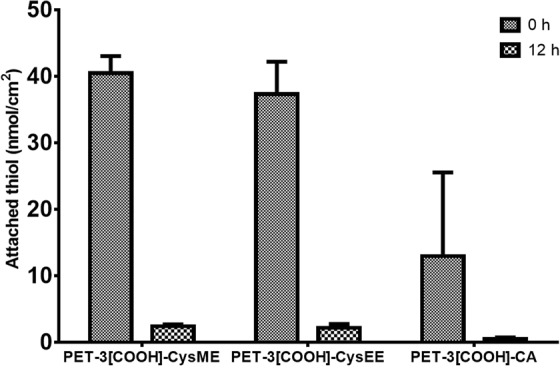
Attachment of different thiol-containing cysteine derivatives [cysteine methyl ester (CysME), cysteine ethyl ester (CysEE), and cysteamine (CA)] on PET-3[COOH] and its stability in PBS (pH = 7.4) after 12 h.

As cysteine and EDC crosslinker are added together during the 1-step immobilisation process, there is a chance of polycysteine formation. This could favour the electrostatic interactions between carboxylated PET and polycysteine. To prevent the formation of polycysteine in solution, cysteine derivatives that lacked free carboxyl groups, viz., cysteine methyl ester (CysME), cysteine ethyl ester (CysEE), and cysteamine (CA) were used. As PET-3[COOH]-Cys showed the maximum immobilised cysteine concentration, these derivatives were immobilised only to PET-3[COOH]. However, these cysteine derivatives also showed a decrease in the free thiol group concentration after being stored in PBS at 37 °C for 12 h (Fig. 3). The mean CysME, CysEE, and CA concentration immobilised on the carboxylated PET surfaces are 40.5, 37.3, 12.9 nmol/cm^2^, respectively. During stability studies, the immobilised CysME, CysEE, and CA concentration reduced to 2.4, 2.2, and 0.5 nmol/cm^2^, respectively. Based on these results, the possible reason for the observed reduction in thiol concentration could be either the formation of disulphide bonds or the electrostatic interactions of free cysteine with the carboxylated PET.

### Understanding the cause of the decrease in immobilised cysteine concentration

**Figure 4:**
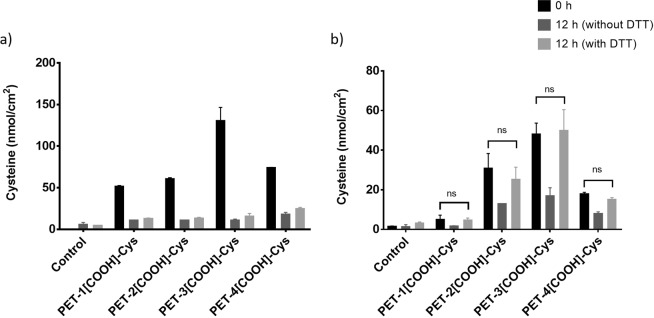
Cysteine immobilisation by (**a**) 1-step EDC crosslinking mechanism and (**b**) 2-step EDC crosslinking mechanism and their stability in PBS (pH = 7.4) at 0 h and 12 h (with and without DTT treatment).

To test if disulphide bonds are formed, the cysteine immobilised PET stored in PBS at 37 °C for 12 h was treated with dithiothreitol (DTT). DTT is a reducing agent that would reduce the disulphide bonds formed between the immobilised cysteine molecules to restore the free thiol groups. Figure 4a shows the immobilised cysteine concentrations on the modified films prepared by 1-step EDC crosslinking. The mean cysteine concentrations immobilised on PET (control), PET-1[COOH]-Cys, PET-2[COOH]-Cys, PET-3[COOH]-Cys, and PET-4[COOH]-Cys are 0, 50.7, 60.1, 130.1, and 73.3 nmol/cm^2^, respectively. After 12 h in PBS at 37 °C, immobilised cysteine concentration on PET (control), PET-1[COOH]-Cys, PET-2[COOH]-Cys, PET-3[COOH]-Cys, and PET-4[COOH]-Cys reduced to 4.9, 10.4, 10.4, 10.4, and 17.3 nmol/cm^2^, respectively. DTT treatment of the samples incubated in PBS for 12 h did not result in any significant change in the immobilised cysteine concentration. This indicates that the cysteine immobilisation through 1-step EDC crosslinking mechanism results in electrostatic interactions based on surface carboxyl density between the cysteine and PET-[COOH] surfaces rather than the formation of stable amide bonds.

As covalent bonds are not formed in the 1-step crosslinking, 2-step EDC crosslinking mechanism was explored. The 2-step crosslinking mechanism uses EDC/NHS for activating the carboxyl groups initially on the PET-[COOH] to form a stable intermediate, which then treated with cysteine should lead to the formation of amide bonds. Figure 4b shows the cysteine concentrations on PET-[COOH]-Cys films prepared by 2-step EDC/NHS crosslinking. The mean cysteine concentrations immobilised on PET (control), PET-1[COOH]-Cys, PET-2[COOH]-Cys, PET-3[COOH]-Cys, and PET-4[COOH]-Cys are 1.2, 4.8, 30.6, 47.7, and 17.6 nmol/cm^2^, respectively. After 12 h in PBS, there was a significant decrease in immobilised cysteine concentration. After DTT treatment of the samples incubated for 12 h, the mean immobilised cysteine concentration on PET (control), PET-1[COOH]-Cys, PET-2[COOH]-Cys, PET-3[COOH]-Cys, and PET-4[COOH]-Cys recovered to values that are comparable to the freshly prepared PET-[COOH]-Cys films.

### Surface characterisation of PET-3[COOH]-Cys

**Figure 5:**
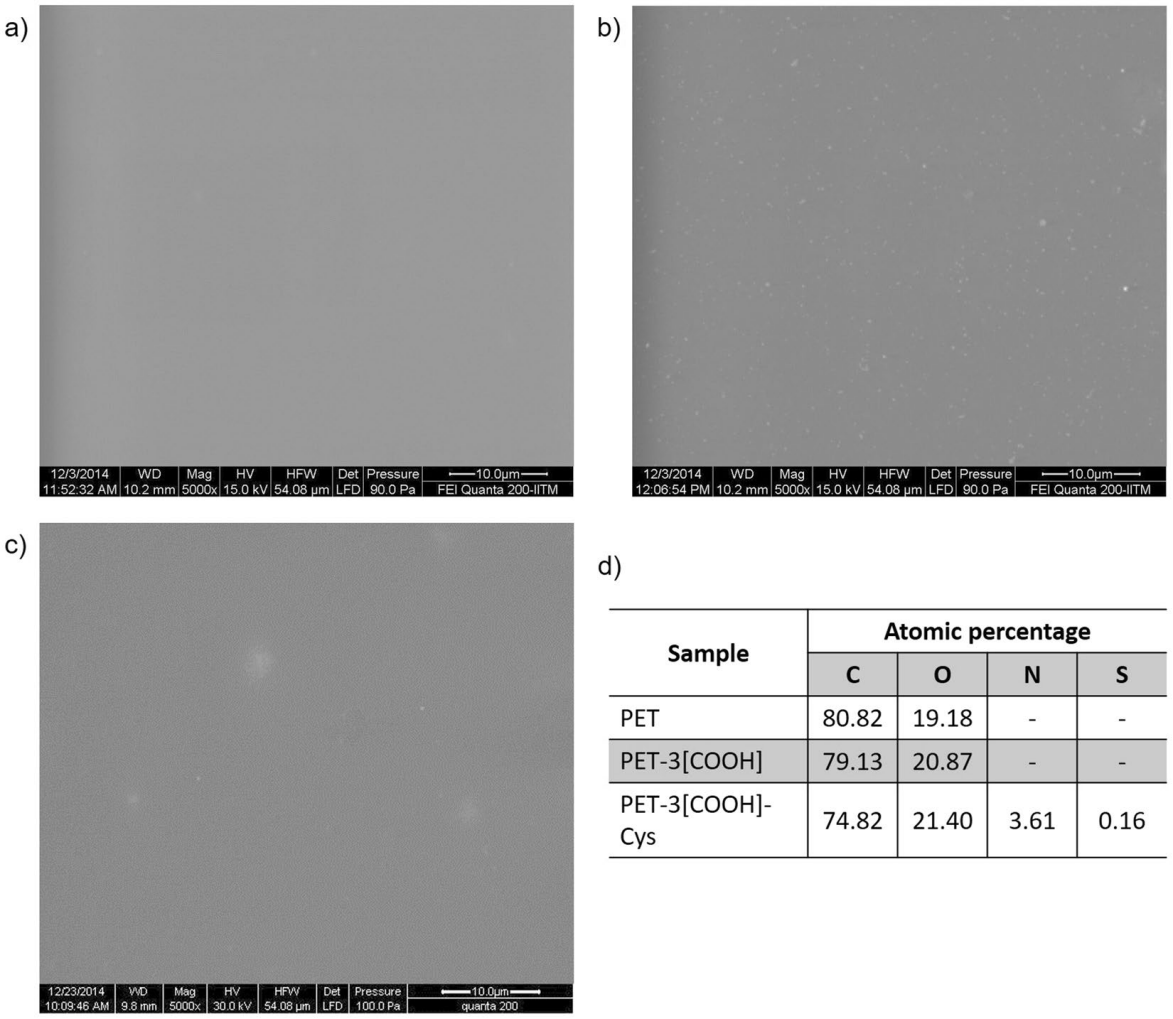
Surface morphology of (**a**) PET, (**b**) PET-3[COOH], (**c**) PET-3[COOH]-Cys, (**d**) Chemical composition of the surfaces estimated by energy dispersive X-ray spectroscopy (EDAX).

To evaluate the potential of cysteine modified surfaces in biomedical applications, it is important to characterise the modified material. Hence, PET-3[COOH]-Cys prepared by the 2-step EDC/NHS crosslinking, which shows the highest cysteine concentration that is covalently attached, was characterised. Surface characterisation of PET-3[COOH]-Cys films was performed using SEM, EDAX and water contact angle measurement. SEM images of the PET-3[COOH]-Cys confirm that carboxylation and cysteine attachment did not cause any significant changes to the surface morphology when compared to unmodified PET (Fig. 5). These images suggest that the carboxylation technique and the cysteine immobilisation procedure used did not damage the polymer surface. EDAX analysis shows the atomic percentage of elements in the unmodified and modified PET surfaces. Unmodified PET in the pristine state showed 80.82% carbon and 19.18% oxygen. The introduction of carboxyl groups on the surface of PET-3[COOH] increased the O % to 20.87. Immobilisation of cysteine on the PET-3[COOH]-Cys surfaces showed the percentages of C, O, N, and S as 74.82, 21.40, 3.61 and 0.16%, respectively. Nitrogen and sulphur content seen on the PET-3[COOH]-Cys confirms the immobilisation of cysteine to PET-3[COOH]. The water contact angle of the PET, PET-3[COOH] and PET-3[COOH]-Cys are 77.1, 53.9, and 55.3°, respectively. Both carboxylated and PET-3[COOH]-Cys have almost similar hydrophilicity suggesting that the wettability was not affected by the cysteine immobilisation.

### Protein adsorption studies

**Figure 6:**
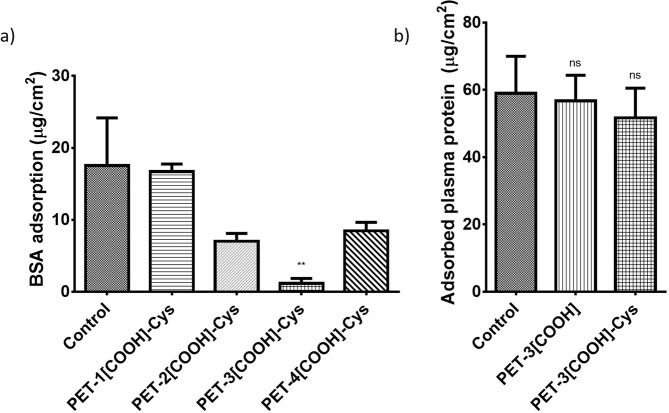
Protein adsorption on unmodified and modified PET surfaces (**a**) Bovine serum albumin adsorption studies (**b**) Plasma protein adsorption studies.

Bovine serum albumin (BSA) was used as a model protein to determine the amount of protein adsorption on the film surface (Fig. 6a). The mean BSA adsorption on PET (control), PET-1[COOH]-Cys, PET-2[COOH]-Cys, PET-3[COOH]-Cys, and PET-4[COOH]-Cys are 17.6, 16.7, 7.1, 1.2, and 8.5 µg/cm^2^, respectively. The results observed on protein adsorption of the PET-[COOH]-Cys were similar to that of the respective carboxylated PET such that PET-1[COOH], PET-2[COOH], PET-3[COOH], and PET-4[COOH] are 16.7, 7.1, 1.9, and 10.2 µg/cm^2^, respectively^29^. Moreover, the PET-3[COOH] and PET-3[COOH]-Cys are the only samples showing a significant reduction in BSA adsorption.

The adsorbed protein fractions from the platelet-poor plasma (PPP) were estimated similarly for PET, PET-3[COOH] and PET-3[COOH]-Cys surfaces (Fig. 6b). The values of the adsorbed protein on unmodified PET (control), PET-3[COOH], and PET-3[COOH]-Cys are 58.9, 56.8 and 51.7 μg/cm^2^, respectively. The plasma proteins adsorbed on the unmodified PET, maximum carboxylated PET and the cysteine immobilised PET surfaces were not statistically different.

### *In vitro* haemocompatibility

**Figure 7:**
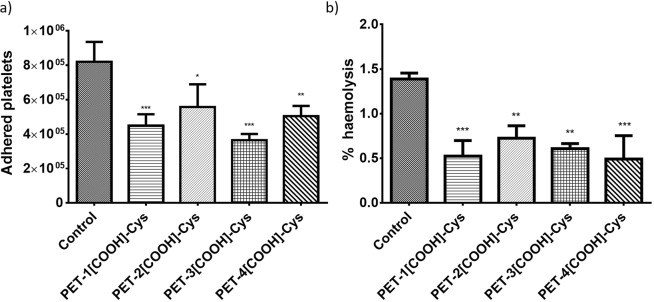
*In vitro* haemocompatibility studies of control and cysteine immobilised PET (PET-[COOH]-Cys) (**a**) quantification of platelet adhesion by LDH assay, (**b**) haemolysis.

Haemocompatibility of the cysteine modified PET surfaces was evaluated using platelet adhesion study and %haemolysis analysis. In platelet adhesion study using LDH assay, platelet-rich plasma (PRP) was incubated with unmodified and modified PET. Figure 7a shows the platelet adhesion on the different PET films. The mean number of platelets adhered on PET (control), PET-1[COOH]-Cys, PET-2[COOH]-Cys, PET-3[COOH]-Cys, PET-4[COOH]-Cys are 8.2, 4.5, 5.6, 3.6, and 5.0 × 10^5^, respectively. However, the platelet adhesion on PET-[COOH] and PET-[COOH]-Cys exhibited almost similar values without any statistically significant difference. The mean number of platelets adhered on PET-1[COOH], PET-2[COOH], PET-3[COOH], and PET-4[COOH] were 3.9, 6.7, 1.6, and 7.4 × 10^5^, respectively^29^.

For the haemolysis studies, all the modified PET samples showed a significant reduction in the %haemolytic index irrespective of the cysteine attachment (Fig. 7b). The mean %haemolysis index for PET (control), PET-1[COOH]-Cys, PET-2[COOH]-Cys, PET-3[COOH]-Cys, PET-4[COOH]-Cys are 1.4, 0.5, 0.7, 0.61, and 0.5%, respectively. Carboxylated PET surfaces also exhibited similar values with PET-1[COOH], PET-2[COOH], PET-3[COOH], and PET-4[COOH] 0.7, 0.6, 0.5, and 0.5%, respectively^29^.

Whole blood studies show the thrombus formation on the unmodified and modified PET surfaces. This was analysed qualitatively using SEM images (Fig. S1).

## Discussion

Nitric oxide, being one of the early thromboregulators, is a promising candidate for developing anti-thrombogenic materials for biomedical applications^18^. Using low molecular weight thiols as a mediator for utilising endogenous NO can potentially provide a sustained supply of NO surrounding the material surface. Here, we have immobilised cysteine on different carboxylated PET surfaces to have better control of NO release and improve the haemocompatibility of the PET surface. Having controlled the amount of carboxylation using the four different methods, the effect of cysteine immobilisation on these four carboxylated PET surfaces were identified. Ellman’s assay showed that the immobilisation of the cysteine on the PET-[COOH] surfaces depends on the surface carboxyl density. Further study of NO release through cysteine by Griess assay also follows the same pattern. PET-3[COOH] having the maximum carboxyl density has the maximum cysteine attachment with maximum NO release through transnitrosation reaction. NO release regulated using different concentrations of immobilised cysteine has a vast potential since NO-mediated effects are concentration-dependent^30–32^.

**Figure 8:**
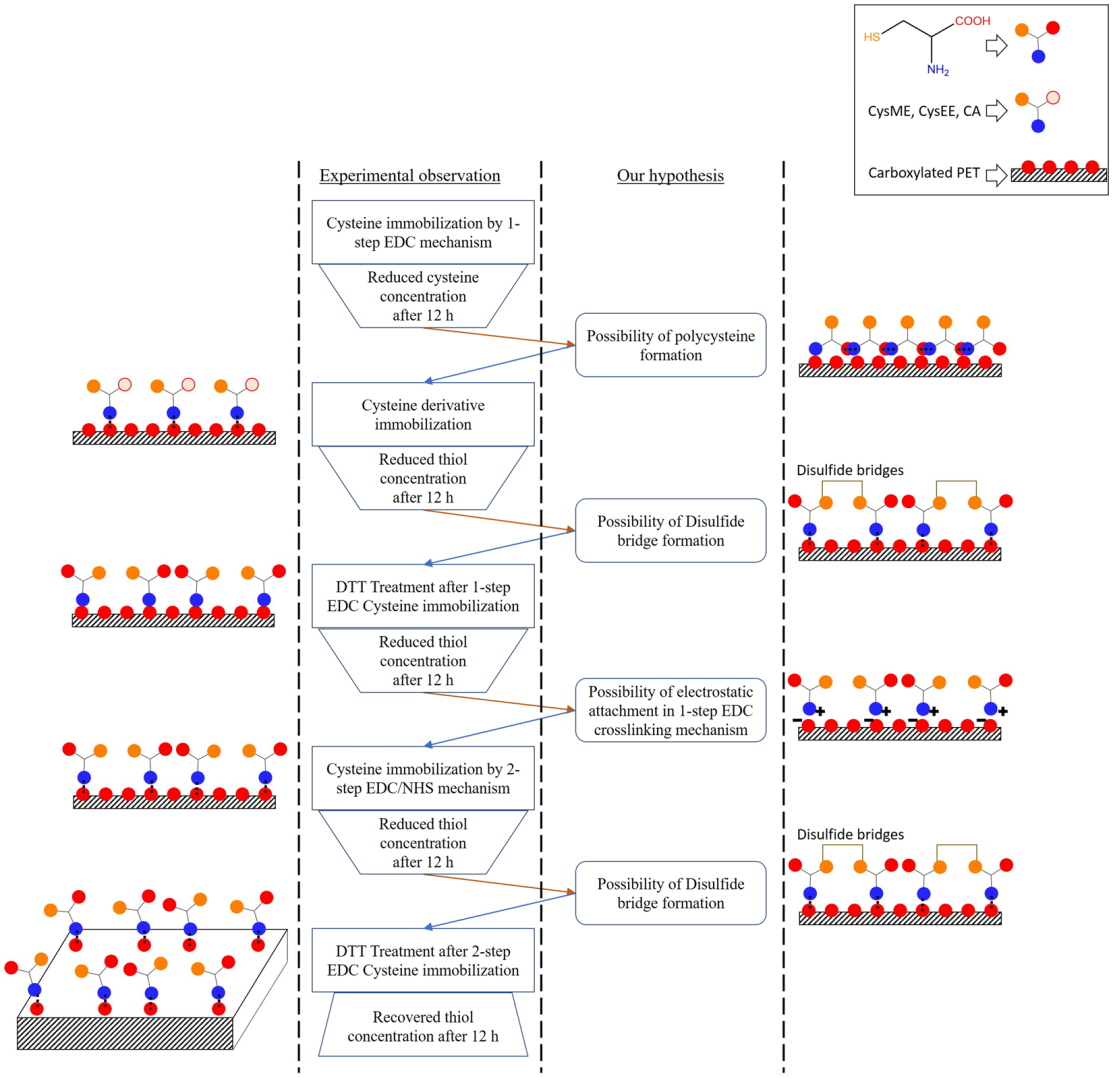
Schematic representation of the outline in troubleshooting the reduction in stability of the immobilised cysteine on the carboxylated PET.

One of the potential advantages of using cysteine is the continuous release of NO from endogenous nitrosothiols over the area of the implant surface to inhibit platelet adhesion and activation. To achieve this, the attached molecule should be stable over a period of time. However, the stability studies showed that the free thiol groups were lost within 12 h of incubation in PBS at 37 °C. One of the possible reasons for the observed reduction in thiol group concentration is the loss of immobilised cysteine from the surface. Cysteine might be lost from the surfaces if it is weakly attached to the carboxylated surfaces instead of forming stable amide bonds through EDC crosslinking. Muthuvijayan *et al*. had suggested the possibility of thioester formation between the thiol group of cysteine and the carboxyl group of PET-[COOH], which could lead to the loss of immobilised cysteine^28^. However, as we are using Ellman’s assay for quantifying cysteine, the free thiol groups are used for measuring cysteine concentration. Hence, we can rule out the formation of thioester bonds. We tested a series of hypothesis for understanding the reason for the reduction in immobilised cysteine (Fig. 8).

One of the possible reasons for weak interactions could be the formation of polycysteine during the 1-step EDC crosslinking mechanism. To prevent the formation of polycysteine, and the subsequent weak interaction, we immobilised three carboxyl-protected cysteine derivatives (CysME, CysEE, CA) to the carboxylated PET surfaces. However, the carboxyl protected cysteine derivatives also exhibited loss of thiol concentration when incubated in PBS for 12 h. The observed reduction removed the possibility of electrostatic interactions due to the formation of polycysteine.

Next, we tested if disulphide bonds were formed between the immobilised cysteine molecules. If the reduction in immobilised cysteine concentration was due to disulphide bond formation, the free thiol groups could be restored by simply reducing these bonds. If cysteine had formed stable amide bonds with carboxylated PET, the thiol concentration after reduction would be comparable to the initial thiol concentration. If the observed reduction in cysteine concentration were due to loss of cysteine, it would imply that cysteine did not form covalent linkages with carboxylated PET. Instead, cysteine was weakly adhered. Hence, if the cysteine immobilisation were through weak interactions, the thiol concentration would not change after reduction. To test this hypothesis, cysteine modified PET that was incubated in PBS for 12 h was treated with DTT. DTT is a potent reducing agent that can break any disulphide bridges formed between the thiol groups of the immobilised cysteine. Hence, comparing the cysteine concentrations of the PET-[COOH]-Cys stored in PBS at 37 °C for 12 h before and after DTT treatment would help in understanding the reason for the observed reduction in cysteine concentration.

DTT treatment of the cysteine immobilised PET prepared using the 1-step EDC crosslinking mechanism did not show a recovery of the immobilised cysteine concentration, which eliminates the possibility of disulphide bond formation in this process. These results point to cysteine not being covalently attached in the 1-step EDC crosslinking mechanism. Hence, 2-step EDC/NHS crosslinking was performed for the immobilisation. The cysteine concentrations on the PET-[COOH]-Cys films prepared by 2-step EDC crosslinking mechanism also decreased when stored in PBS. However, when the stored PET-[COOH]-Cys was treated with DTT, the measured cysteine concentration recovered and reached a value that was comparable to the initial concentration of the freshly prepared PET-[COOH]-Cys. This indicates that in 2-step EDC crosslinking mechanism, cysteine is not lost from the surface indicating that cysteine is not weakly adhered and covalent bonds are formed. Unfortunately, the free thiol groups are lost over time due to the disulphide bridge formation. Having a complex polymer base, there is a high probability of forming disulphide bonds due to the proximity of thiol groups on the modified surfaces. Ironically, the free sulfhydryl group needed for the continuous release of NO from endogenous nitrosothiols is lost due to this disulphide bridge formation. This implies that the cysteine modified PET could be suitable for short-term applications, but might not be very effective for long-term implants.

However, to study if cysteine immobilisation can help in short-term applications, the PET-3[COOH]-Cys, which was prepared using the 2-step EDC crosslinking and showed the maximum cysteine concentration, was characterised. Surface characterisation using SEM and contact angle shows that the cysteine modified PET has properties that are comparable to carboxylated PET. EDAX showed the change in the surface elemental composition, confirming the successful immobilisation of cysteine on carboxylated PET. For protein adsorption studies, the cysteine immobilised PET was comparable to the carboxylated PET. The electronegative surface carboxyl groups have reduced BSA adsorption because, at physiological pH, the effective zeta potential of BSA is negative^33^. Also, a hydration layer could have formed due to the layer of carboxyl groups on the modified surfaces. This would result in an energy barrier that inhibits random protein adsorption^34^. As PET-[COOH]-Cys also exhibits similar protein adsorption as PET-[COOH], it suggests that protein adsorption might be due to the surface properties such as wettability, charge, and topography. Carboxyl groups available on the immobilised cysteine might result in similar protein adsorption. However, there is no statistically significant reduction of adsorbed plasma proteins on the PET-3[COOH]-Cys and PET-3[COOH] surfaces when compared to control PET. This might be due to the non-specific binding of different proteins present in the plasma pool. Although albumin, the major component of the blood proteins, might show reduced adsorption, the other proteins present in the plasma result in comparable protein adsorption on the unmodified, carboxylated, and cysteine immobilised PET surfaces. This implies that cysteine immobilisation has no significant effect on protein adsorption under physiological conditions.

Similarly, the *in vitro* haemocompatibility studies show that the performance of cysteine immobilised PET is comparable to the carboxylated PET. The PET-3[COOH] and PET-3[COOH]-Cys show the least platelet adhesion compared to all other modified methods. The cysteine attached polymer has almost the same effect as that of the carboxylated PET suggesting that the thiol groups were not available for the NO release by the transnitrosation reaction. The formation of the disulphide bridges between the immobilised cysteine molecules could be the reason for the lack of enhanced haemocompatibility on PET-[COOH]-Cys. Cysteine modification on the polymer grafting techniques (PET-2[COOH] and PET-4[COOH]) showed a slight reduction in platelet adhesion compared to the carboxylated PET. This might be due to the gel-like nature of the grafted polymer, which could have restricted the formation of disulphide bridges. These results suggest that factors such as surface wettability, charge, and topography of different methods of carboxylation are involved in platelet adhesion. Cysteine being an essential amino acid, plays no role in the lysis of erythrocytes. However, the surface carboxyl groups tend to decrease the %haemolysis^35^. Hence, %haemolysis of the cysteine modified polymers is similar to that of the carboxylated PET. These results indicate that the properties such as surface charge, hydrophilicity, and topography play a crucial role in the haemocompatibility of cysteine immobilised PET. The thrombus formation in the whole blood studies shows that the control PET is thrombogenic. However, the PET-3[COOH] and the PET-3[COOH]-Cys surfaces showed reduced thrombus formation than the unmodified PET. This corroborates with the platelet adhesion studies showing that the carboxylated and the cysteine immobilised PET surfaces exhibit comparable haemocompatibility properties. As free thiol groups might not have been available for the transnitrosation reaction, cysteine immobilisation did not enhance the haemocompatibility of the material.

In conclusion, we have immobilised cysteine on four different carboxylated PET. The mechanism of covalent immobilisation was understood using different mechanisms of crosslinking. Interestingly, the free thiol group needed for transnitrosation reaction tends to form disulphide bridges with the adjacent cysteine molecule. The lack of free thiol groups of immobilised cysteine limits the potential of these modified surfaces in long-term applications. Further studies on the spatial arrangement of immobilisation will be a constructive way of using cysteine to improve the functionality of PET efficiently.

## Materials and Methods

### Materials

PET films of 100-micron thickness were kindly gifted by Sumilon Polyester Ltd. (India). Cysteine, 5,5′-Dithiobis (2-nitrobenzoic acid) (Ellman’s reagent), Griess reagent (modified) kit, lactate dehydrogenase (LDH) activity assay kit and bicinchoninic acid (BCA) kit were purchased from Sigma-Aldrich. Analytical grade reagents were used for all other experiments unless otherwise specified.

### Different methods of carboxylation

Unmodified PET films (1 × 5 cm^2^) were washed in acetone for 24 h, and vacuum dried. These PET films were used for further experiments to produce carboxylated PET (PET-[COOH). The different methods of surface carboxylation of the PET surface were done as previously described^29^. PET-1[COOH]: formaldehyde + bromoacetic acid treatment; PET-2[COOH]: methacrylic acid grafting; PET-3[COOH]: NaOH hydrolysis + KMnO_4_ oxidation; PET-4[COOH]: oxygen plasma treatment + acrylic acid grafting. All the PET-[COOH] were washed with excess water and stored in a nitrogen environment before any further surface modification.

### Immobilisation of cysteine and optimising the reaction conditions

The cysteine was covalently attached to the carboxylated PET surfaces using a 1-step crosslinking mechanism. This mechanism uses 1-ethyl-3-[3-dimethylaminopropyl] carbodiimide (EDC), a water-soluble carbodiimide as the crosslinker. Briefly, the sample was incubated in 0.1 M phosphate buffer (pH = 6.8) containing 0.1 M EDC and 0.3 M cysteine for 12 h at room temperature. After that, the samples were washed in 1 M sodium chloride and excess of water. Then, the cysteine immobilised PET films (PET-[COOH]-Cys) were dried and stored in a nitrogen glove box for further studies.

### Estimation of cysteine attachment

The cysteine immobilised on the PET-[COOH]-Cys surfaces was estimated by the 5,5′-dithio-bis-(2-nitrobenzoic acid) (DTNB, also known as Ellman’s reagent), which reacts with the free sulfhydryl group^36^. Ellman’s reagent solution was prepared by dissolving 4 mg DTNB in 1 mL of reaction buffer (0.1 M phosphate buffer, pH = 8.0, containing 1 mM EDTA). For each sample of PET-[COOH]-Cys, 2.5 mL of reaction buffer and 50 µL of Ellman’s reagent were added. The samples were incubated at room temperature for 20 min with constant mixing. The absorbance of the solution was measured at 412 nm. The standard is prepared from the known concentrations of cysteine.

### Estimation of nitric oxide release

The amount of nitric oxide (NO) released by the reaction between the thiol group of the cysteine and nitrite was estimated using modified Griess reagent kit^37^. 50 µM of sodium nitrite in 0.5 N hydrochloric acid was supplied as a substrate for NO. The protocol for the kit was followed for the estimation of nitric oxide. Briefly, equal volumes of Griess Reagent and sample were mixed and incubated for 15 min. The absorbance of the solution was measured at 540 nm. The standard is prepared from the known concentrations of nitrite.

### Assessment of thiol group stability on modified PET

To study the stability, PET-[COOH]-Cys was stored in PBS at 37 °C for different time intervals (0, 6, 12, 18, 24, and 30 h). The concentrations of immobilised cysteine on these stored PET-[COOH]-Cys films were measured using Ellman’s assay. Also, the amounts of NO released from these films were quantified using the Greiss assay.

### Attachment of cysteine derivatives on PET-3[COOH] and their thiol group stability

Other cysteine derivatives such as cysteine methyl ester (CysME), cysteine ethyl ester (CysEE), and cysteamine (CA) were also immobilised on the maximum carboxylated PET surface (PET-3[COOH]). The cysteine derivatives were immobilised using the 1-step crosslinking protocol mentioned in the earlier section. The stability of these films modified with cysteine derivatives was studied for the freshly prepared samples and the samples stored in PBS (pH = 7.4) at 37 °C for 12 h.

### DTT treatment of modified PET

PET-[COOH]-Cys films stored in PBS at 37 °C for 12 h, were treated with 10 mM dithiothreitol (DTT) solution for 10 min at room temperature. DTT can reduce disulphide bonds to form free thiol groups^38^. The cysteine concentration on the DTT-treated surfaces was measured using Ellman’s reagent.

### 2-step EDC crosslinking mechanism

Here, the immobilisation of cysteine was done in two steps using EDC/NHS mechanism. The first step involves the activation of carboxyl groups on the PET surfaces by EDC. Briefly, the PET-[COOH] films were treated with 0.1 M EDC and 0.1 M N-hydroxysuccinimide (NHS) in 0.1 M 2-(N-morpholino) ethanesulphonic acid buffer (pH = 5.5, 0.5 M NaCl) for 2 h at room temperature with constant agitation. The second step involves the amide bond formation between the amine group of cysteine and the carboxylated PET surfaces. Briefly, the activated PET-[COOH] films were incubated with 0.3 M cysteine in 0.1 M phosphate buffer (pH = 7.4, 0.15 M NaCl) for 12 h at room temperature with constant agitation. After completion of the reaction, the films were taken out and washed with 1 M NaCl for 30 min and then with an excess amount of deionised water. The stability studies were performed, in association with DTT treatment mentioned in the previous section, on the stored PET-[COOH]-Cys films to reduce disulphide bonds. The cysteine concentration on the DTT-treated surfaces was measured using Ellman’s reagent.

### Surface characterisation

The modified polymers with the maximum cysteine concentration prepared by the 2-step crosslinking mechanism (PET-3[COOH]-Cys) were characterised further to study their surface properties. High-resolution electron micrographs of the unmodified and modified PET surfaces were recorded using a scanning electron microscope (SEM), Quanta 200, FEI. The chemical composition of the unmodified and modified PET surfaces was analysed using energy dispersive X-ray spectroscopy (EDAX). The samples were sputtered with gold before the observation. Static water contact angles were measured on the unmodified and modified PET surfaces using the sessile drop method. The contact angle measurements were carried out in air using a goniometer (DSAII GmbH, KRUSS). Approximately 2 μL of MilliQ water was dropped on the sample surface at room temperature, and the water contact angle was recorded. The surface wettability was determined on different locations on a given sample, and the average of these values was calculated.

### Protein adsorption study

Adsorption of bovine serum albumin (BSA) and plasma protein on the modified and unmodified PET surfaces was quantified. Briefly, PET, PET-[COOH], and PET-[COOH]-Cys films (1 × 1 cm^2^) were pre-treated and equilibrated in phosphate buffer saline (PBS) for 1 h. BSA solution (1.0 mg/mL) was incubated with the samples at 37 °C for 2 h. These samples were thoroughly washed with PBS, followed by rinsing with water. The adsorbed protein was removed from the surfaces by incubating the films in 1% sodium dodecyl sulfate (SDS) solution for 2 h at 37 °C. Then, the samples were sonicated for 20 min to remove the adsorbed BSA completely. The concentration of adsorbed BSA was quantified using the micro BCA protein assay kit. The assay was performed as per the manufacturer’s protocol.

For plasma protein adsorption studies, the freshly collected anticoagulated blood was centrifuged at 3000 g for 5 min at 4 °C to isolate the platelet-poor plasma (PPP). Similar to the BSA adsorption studies, plasma proteins adsorbed on PET, PET-3[COOH] and PET-3[COOH]-Cys were quantified.

### *In vitro* haemocompatibility studies

All the blood-related studies were approved by the Institute Ethics Committee, Indian Institute of Technology Madras (IEC/2017/04/VMV/15). The haemocompatibility experiments were carried out in accordance with the National Ethical Guidelines for Biomedical and Health Research Involving Human Participants, 2017 issued by Indian Council of Medical Research (ICMR). Fresh blood samples were collected from the healthy volunteers after obtaining informed consent following the ICMR regulations. Blood was collected in vacutainer tubes preloaded with 3.8% sodium citrate solution.

*In vitro* platelet adhesion on the samples were studied using LDH assay. The anticoagulated blood was centrifuged at 1500 rpm for 15 min at 4 °C to isolate the platelet-rich plasma (PRP). PET, PET-[COOH], and PET-[COOH]-Cys films (1 × 1 cm^2^) were pre-treated and equilibrated in PBS for 1 h. Then, the films were placed in a 24-well tissue culture plate and incubated with 200 µL of the diluted PRP (PRP: PBS, 1:1 v/v) at 37 °C for 1 h under static conditions. After platelet adhesion, all the samples were gently washed with PBS. LDH assay kit was used to quantify the number of adhered platelets on the sample surfaces. A standard curve that was plotted using serially diluted samples with a known number of platelets (counted using haemocytometer) was used to estimate the unknown concentrations.

Haemolysis assay was used to identify the effect of material on the lysis of erythrocytes in blood. 4 mL of whole blood was diluted in 5 mL of normal saline solution (0.9% NaCl) for the protocol. Unmodified and modified films (1 × 1 cm^2^) were immersed in 5 mL saline solution for 30 min at 37 °C. These equilibrated films were incubated with 100 µL of the diluted blood at 37 °C for 2 h. After incubation, the samples were centrifuged at 1500 rpm for 10 min. The supernatant was isolated and estimated for erythrocyte lysis. The absorbance (O.D.) of the released haemoglobin was measured at 545 nm. A mixture of 100 µL of blood in 5 mL of deionised water was used as the positive control. A mixture of 100 µL of blood in 5 mL saline was used as the negative control. The following equation was used to calculate %haemolysis,

$${\rm{ \% }}\,Haemolysis=\frac{O.D{.}_{(sample)}-O.D{.}_{(Negativecontrol)}}{O.D{.}_{(Positivecontrol)}-O.D{.}_{(Negativecontrol)}}\times 100$$

The whole blood studies were done using the freshly collected anticoagulated blood. The samples PET, PET-3[COOH] and PET-3[COOH]-Cys were incubated with the whole blood for 2 h at 37 °C. After incubation, all the samples were gently washed with PBS to remove the loosely adhered blood components and fixed using 2.5% glutaraldehyde for 1 h at 37 °C. Then, the samples were dehydrated with ethanol-water mixtures of increasing concentrations (50, 60, 70, 80, 90, and 100 ethanol vol %). The samples were observed under an SEM.

### Statistical analysis

All the experiments were performed in triplicates and expressed as a mean ± standard deviation. The statistical analysis was performed using the ANOVA followed by Bonferroni’s test on GraphPad Prism (version 5.0; GraphPad Software Inc. San Diego CA, California, USA). The values of *p < 0.05, **p < 0.01, and ***p < 0.001 were considered as statistically significant.

## Supplementary information

Supplementary Information

## References

[1] Moncada S, Palmer RM, Higgs EA (1991). Nitric oxide: physiology, pathophysiology, and pharmacology. Pharmacological reviews.

[2] Kannan MS, Guiang S, Johnson DE (1998). Nitric oxide: biological role and clinical uses. Indian journal of pediatrics.

[3] Radomski MW, Palmer RM, Moncada S (1991). Modulation of platelet aggregation by an L-arginine-nitric oxide pathway. Trends in pharmacological sciences.

[4] de Graaf JC (1992). Nitric oxide functions as an inhibitor of platelet adhesion under flow conditions. Circulation.

[5] Scott-Burden T, Vanhoutte PM (1994). Regulation of smooth muscle cell growth by endothelium-derived factors. Texas Heart Institute journal.

[6] Nakaki T, Nakayama M, Kato R (1990). Inhibition by nitric oxide and nitric oxide-producing vasodilators of DNA synthesis in vascular smooth muscle cells. European journal of pharmacology.

[7] Schairer DO, Chouake JS, Nosanchuk JD, Friedman AJ (2012). The potential of nitric oxide releasing therapies as antimicrobial agents. Virulence.

[8] Bohl KS, West JL (2000). Nitric oxide-generating polymers reduce platelet adhesion and smooth muscle cell proliferation. Biomaterials.

[9] Nablo BJ, Prichard HL, Butler RD, Klitzman B, Schoenfisch MH (2005). Inhibition of implant-associated infections via nitric oxide release. Biomaterials.

[10] Kelm M, Schrader J (1988). Nitric oxide release from the isolated guinea pig heart. European journal of pharmacology.

[11] Frost MC, Reynolds MM, Meyerhoff ME (2005). Polymers incorporating nitric oxide releasing/generating substances for improved biocompatibility of blood-contacting medical devices. Biomaterials.

[12] Jen MC, Serrano MC, van Lith R, Ameer GA (2012). Polymer-Based Nitric Oxide Therapies: Recent Insights for Biomedical Applications. Advanced functional materials.

[13] Smith DJ (1996). Nitric oxide-releasing polymers containing the [N(O)NO]- group. Journal of medicinal chemistry.

[14] Pulfer SK, Ott D, Smith DJ (1997). Incorporation of nitric oxide-releasing crosslinked polyethyleneimine microspheres into vascular grafts. Journal of biomedical materials research.

[15] Zhang H (2003). Nitric oxide-releasing fumed silica particles: synthesis, characterization, and biomedical application. Journal of the American Chemical Society.

[16] Riccio DA (2009). Nitric oxide-releasing S-nitrosothiol-modified xerogels. Biomaterials.

[17] Frost MC, Meyerhoff ME (2004). Controlled photoinitiated release of nitric oxide from polymer films containing S-nitroso-N-acetyl-DL-penicillamine derivatized fumed silica filler. Journal of the American Chemical Society.

[18] Yang Y, Qi PK, Yang ZL, Huang N (2015). Nitric oxide based strategies for applications of biomedical devices. Biosurface and Biotribology.

[19] Vaughn MW, Kuo L, Liao JC (1998). Estimation of nitric oxide production and reaction rates in tissue by use of a mathematical model. The American journal of physiology.

[20] Hogg N (2000). Biological chemistry and clinical potential of S-nitrosothiols. Free radical biology & medicine.

[21] Williams DL (2003). A chemist’s view of the nitric oxide story. Organic & biomolecular chemistry.

[22] Meyer DJ, Kramer H, Ozer N, Coles B, Ketterer B (1994). Kinetics and equilibria of S-nitrosothiol-thiol exchange between glutathione, cysteine, penicillamines and serum albumin. FEBS letters.

[23] Scharfstein JS (1994). *In vivo* transfer of nitric oxide between a plasma protein-bound reservoir and low molecular weight thiols. The Journal of clinical investigation.

[24] Duan X, Lewis RS (2002). Improved haemocompatibility of cysteine-modified polymers via endogenous nitric oxide. Biomaterials.

[25] Gappa-Fahlenkamp H, Duan X, Lewis RS (2004). Analysis of immobilized L-cysteine on polymers. Journal of biomedical materials research. Part A.

[26] Irena G, Jolanta B, Karolina Z (2009). Chemical modification of poly(ethylene terephthalate) and immobilization of the selected enzymes on the modified film. Appl Surf Sci.

[27] Reddy N, Reddy R, Jiang Q (2015). Crosslinking biopolymers for biomedical applications. Trends in biotechnology.

[28] Muthuvijayan V, Gu J, Lewis RS (2009). Analysis of functionalized polyethylene terephthalate with immobilized NTPDase and cysteine. Acta biomaterialia.

[29] Ramachandran B, Chakraborty S, Dixit M, Muthuvijayan V (2018). A comparative study of polyethylene terephthalate surface carboxylation techniques: Characterization, *in vitro* haemocompatibility and endothelialization. Reactive and Functional Polymers.

[30] Palmer RMJ, Ferrige AG, Moncada S (1987). Nitric oxide release accounts for the biological activity of endothelium-derived relaxing factor. Nature.

[31] Wang GR, Zhu Y, Halushka PV, Lincoln TM, Mendelsohn ME (1998). Mechanism of platelet inhibition by nitric oxide: *in vivo* phosphorylation of thromboxane receptor by cyclic GMP-dependent protein kinase. Proc Natl Acad Sci USA.

[32] Marjanovic JA, Li Z, Stojanovic A, Du X (2005). Stimulatory Roles of Nitric-oxide Synthase 3 and Guanylyl Cyclase in Platelet Activation. Journal of Biological Chemistry.

[33] Jachimska B, Pajor A (2012). Physico-chemical characterization of bovine serum albumin in solution and as deposited on surfaces. Bioelectrochemistry.

[34] Chen S, Li L, Zhao C, Zheng J (2010). Surface hydration: Principles and applications toward low-fouling/nonfouling biomaterials. Polymer.

[35] Xiao K (2011). The effect of surface charge on *in vivo* biodistribution of PEG-oligocholic acid based micellar nanoparticles. Biomaterials.

[36] Riddles PW, Blakeley RL, Zerner B (1979). Ellman’s reagent: 5,5′-dithiobis(2-nitrobenzoic acid)–a reexamination. Analytical biochemistry.

[37] Green LC (1982). Analysis of nitrate, nitrite, and [15N]nitrate in biological fluids. Analytical biochemistry.

[38] Cleland WW (2002). Dithiothreitol, a New Protective Reagent for SH Groups*. Biochemistry.

