# Supplementary material for: Cysteine immobilisation on the polyethylene terephthalate surfaces and its effect on the haemocompatibility
Source: Sci Rep. 2019 Nov 13;9:16694. doi: 10.1038/s41598-019-53108-2 (PMC6853964; doi:10.1038/s41598-019-53108-2)
Supplement: Supplementary file 1 — Supplementary Information [file 41598_2019_53108_MOESM1_ESM.pdf]

Supplementary information

Cysteine immobilisation on the polyethylene terephthalate surfaces and its effect on the haemocompatibility

Balaji Ramachandran[a] and Vignesh Muthuvijayan [a]*

[a]*Department of Biotechnology, Bhupat and Jyoti Mehta School of Biosciences, Indian Institute of Technology Madras, India*

*Correspondence to: Vignesh Muthuvijayan, Department of Biotechnology, Indian Institute of Technology Madras, Chennai 600036, India. E-mail id: vigneshm@iitm.ac.in. Tel no: 91-44-2257-4123

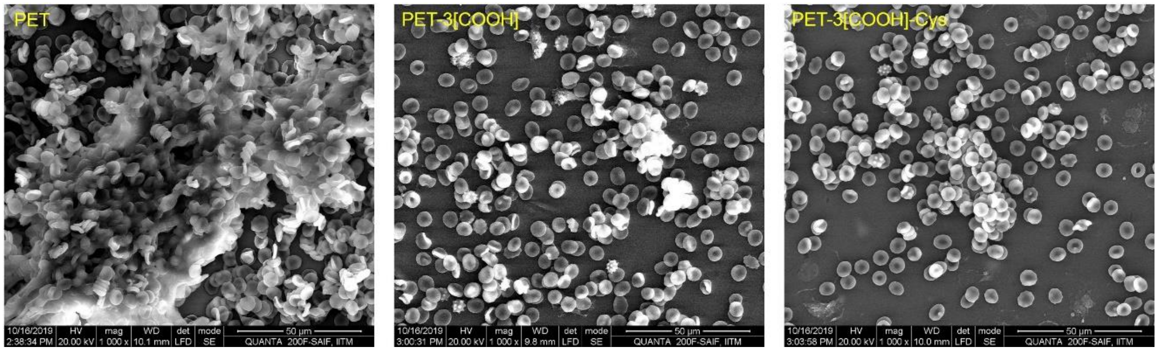

Figure S1. SEM images of adhered whole blood on PET, PET-3[COOH] and PET-3[COOH]-Cys surfaces.
